# Impact of central obesity on prognostic outcome of triple negative breast cancer in Chinese women

**DOI:** 10.1186/s40064-016-2200-y

**Published:** 2016-05-11

**Authors:** Hong-liang Chen, Ang Ding, Mao-li Wang

**Affiliations:** Breast Surgery Department, Obstetrics and Gynecology Hospital of Fudan University, Shanghai, 200011 China

**Keywords:** Obesity, Central obesity, Triple negative breast cancer, Prognosis

## Abstract

**Purpose:**

To evaluate the prognostic effect of central obesity on triple negative breast cancer (TNBC).

**Methods:**

206 TNBC patients treated from June 2006 to June 2015 were enrolled retrospectively. Body mass index (BMI) ≥25 kg/m^2^ was the standard of obesity and waist circumference ≥80 cm was the standard of central obesity. Patient and tumor characteristics were compared between obesity categories. Survival differences between obesity categories were assessed with log-rank test in the univariate analysis and prognostic factors were then investigated by Cox regression analysis.

**Results:**

81 cases were with obesity (39.3 %). 71 cases were with central obesity (34.5 %). Patients with obesity or central obesity tended to be older (P = 0.022 for obesity; P = 0.013 for central obesity) and to have larger tumor size (P = 0.027 for obesity; P = 0.027 for central obesity). By Cox regression analysis, central obesity (DFS: HR 1.759; 95 % CI 1.009–3.065; P = 0.046. OS: HR 2.297; 95 % CI 1.184–4.456; P = 0.014) was identified as an independent prognostic factor. For central obesity with BMI ≥ 25 kg/m^2^, the prognostic effect was more apparent (DFS: HR 1.845; 95 % CI 1.059–3.212; P = 0.031. OS: HR 2.377; 95 % CI 1.230–4.593; P = 0.010).

**Conclusion:**

Central obesity, especially with high BMI, was an independent prognostic factor for TNBC.

## Background

Triple negative breast cancer (TNBC) is defined by the absence of estrogen receptor (ER), progesterone receptor (PR), and human epidermal growth factor receptor-2 (HER-2) overexpression, which accounts for 15–20 % of breast cancer patients (Perou et al. 2000). It is characterized by occurrence at young age and a high propensity of early metastasis to distant visceral organs (Chen and Ding 2015). Women with TNBC have worse prognostic outcomes compared with those with other subtypes (Dent et al. 2007). Given the lack of targeted therapy and limited treatment options, researchers have paid much attention on some modifiable factors associated with the prognosis of TNBC.

Obesity is now a common health problem worldwide. It is a lifestyle risk factor associated with not only high risk of cardiovascular and metabolic disease, but also with high incidence and poor prognosis of many malignant tumors (Ryan and Kushner 2010; Renehan et al. 2008). A growing body of literature indicates that metabolism syndrome is related closely with the development and progression of TNBC (Davis and Kaklamani 2012; Maiti et al. 2010). Obesity, especially central obesity plays a central role in metabolism syndrome. It is well acknowledged that obesity is associated with a worse clinical outcome in breast cancer patients (Kroenke et al. 2005; Petrelli et al. 2002; Loi et al. 2005; Abrahamson et al. 2006). But most researches included breast cancer cases of all subtypes or only cases with positive hormone receptors. Limited researches have evaluated the associations of obesity at diagnosis on TNBC prognosis, and the findings are mixed (Ademuyiwa et al. 2011; Sparano et al. 2012; Mowad et al. 2013; Tait et al. 2014; Dawood et al. 2012; Turkoz et al. 2013; Hao et al. 2015). Meanwhile as far as our knowledge, there is no literature specifically focused on the significance of central obesity among TNBC patients.

## Purpose

The purpose of this study is to retrospectively investigate the effects of central obesity at the time of breast cancer diagnosis on recurrence and mortality among women with TNBC who were treated at our hospital.

## Methods

### Patients

We retrospectively enrolled 206 TNBC patients who were treated at the Department of Breast Surgery of Obstetrics and Gynecology Hospital of Fudan University from June 2006 to June 2015. The status of the estrogen receptor (ER), progesterone receptor (PR), and human epidermal growth factor receptor-2 (HER-2) was determined by immunohistochemical (IHC) staining. TNBC was defined by ER positivity and PR positivity less than 1 % of tumor cells with positive nuclear staining and HER-2 status (−) or (1+) by IHC or lack of gene amplification confirmed by florescent in situ hybridization (FISH).

This study was approved and exempted from patient permission by Institutional Review Boards. All patients received standard treatment, including mastectomy or breast-conserving surgery (BCS) plus axillary lymph node dissection (ALND)/sentinel lymph node biopsy (SLNB), adjuvant/neoadjuvant chemotherapy composed of anthracyclines and/or taxanes followed by radiotherapy (if required). All patients with positive lymph node metastasis received ALND. Patients were excluded for the following reasons: male gender, in situ lesion, curative resection not conducted, and distant metastasis confirmed before surgery.

### Obesity standards

We identified body mass index (BMI) ≥25 kg/m^2^ as the standard of obesity and waist circumference (WC) ≥ 80 cm for women as the standard of central obesity according to the recommendation of the international diabetics federation. BMI is computed by dividing the weight in kilograms by the square of the height in meters.

### Follow-up

Overall survival (OS) was calculated as the time from diagnosis to death or last follow-up and disease-free survival (DFS) was calculated as the time from diagnosis to first recurrence/metastasis or last follow-up. Those without any evidence of relapse were censored at the last date on which they were known to be alive. Patients who were lost to follow-up were censored at the date of their last follow-up. All recurrences were diagnosed by either clinical or radiological examinations. Follow-up information regarding tumor relapse and survival status was available through outpatient departmental records and personal contact with the patients via mail and telephone calls until Jan 2016.

### Statistics

Patient and tumor characteristics were compared between obesity categories using an independent sample *t* test for continuous variables and the Pearson Chi square test for categorical variables. Fisher’s exact test was used when needed. The Kaplan–Meier method was used to generate survival curves and differences between obesity categories were assessed with the log-rank test. All variables with statistical significance in the univariate analysis were investigated by multivariate analysis to compare survival outcomes among obesity categories. Adjusted hazard ratios (HR) with 95 % confidence intervals (95 % CI) were calculated using Cox proportional hazards model. All the statistical analysis was performed using SPSS 19.0 software package (SPSS, Chicago, IL, USA). Two-sided P < 0.05 was considered statistically significant.

## Results

### Baseline information and patient characteristics by obesity categories

206 patients with AJCC stage I to III TNBC who had baseline weight and height recorded were identified. The median age for the entire cohort was 48.5 years (range 27–73 years). BMI ranged from 18.7 to 31.1 kg/m^2^ (median 23.8 kg/m^2^). According to the standard of obesity as BMI ≥ 25 kg/m^2^, 81 cases were with obesity (39.3 %) and according to the standard of central obesity as WC ≥ 80 cm, 71 cases were with central obesity (34.5 %). Among the 71 cases, 23 cases had diabetics, 32 cases had hypertension, and 25 cases had hyperlipidemia. It was observed that among TNBC patients with central obesity, 67 cases had their BMI ≥ 25 kg/m^2^. The other 4 cases with central obesity but BMI < 25 kg/m^2^ were relatively tall and had no such metabolic diseases.

The majority of the study population who received adjuvant/neoadjuvant chemotherapy received an anthracycline-based regimen (96.1 %); doxorubicin plus cyclophosphamide followed by a taxane was the most frequent regimen administered.

Distributions of patient characteristics by obesity categories were tabulated in Table 1. Patients with obesity or central obesity tended to be older (BMI ≥ 25 kg/m^2^: 51.8 vs 48.5 years, *P* = 0.022; WC ≥ 80 cm: 52.3 vs 48.5 years, *P* = 0.013), and to have larger tumor size (BMI ≥ 25 kg/m^2^: 3.34 vs 2.71 cm, *P* = 0.027; WC ≥ 80 cm: 3.38 vs 2.73 cm, *P* = 0.027) and higher proportion of pT_2_ and pT_3_ stage (BMI ≥ 25 kg/m^2^: *P* = 0.031; WC ≥ 80 cm: *P* = 0.044). There was no significant difference among obesity or central obesity groups with regard to menopausal status, histologic grade, pN stage, lymphovascular invasion (LVI), and surgery type.

**Table 1:** Clinico-pathological characteristics of TNBC patients among obesity categories

	Obesity			Central obesity		
	BMI ≥ 25 kg/m^2^ (%)	BMI < 25 kg/m^2^ (%)	*P*	WC ≥ 80 cm (%)	WC < 80 cm (%)	*P*
Menopausal status			0.130			0.245
Pre-menopausal	40 (49.4)	73 (58.4)		35 (49.3)	78 (57.8)	
Post-menopausal	41 (50.6)	52 (41.6)		36 (50.7)	57 (42.2)	
pT stage			0.031			0.044
pT_1_	21 (25.9)	55 (44.0)		18 (25.4)	58 (43.0)	
pT_2_	52 (64.2)	60 (48.0)		46 (64.8)	66 (48.9)	
pT_3_	8 (9.9)	10 (8.0)		7 (9.9)	11 (8.1)	
pN stage			0.446			0.987
pN_0_	43 (53.1)	78 (62.4)		41 (57.7)	80 (59.3)	
pN_1_	21 (25.9)	29 (23.2)		17 (23.9)	33 (24.4)	
pN_2_	14 (17.3)	13 (10.4)		10 (14.1)	17 (12.6)	
pN_3_	3 (3.7)	5 (4.0)		3 (4.2)	5 (3.7)	
LVI			0.623			0.140
Positive	29 (35.8)	49 (39.2)		22 (31.0)	56 (41.5)	
Negative	52 (64.2)	76 (60.8)		49 (69.0)	79 (58.5)	
Histologic grade			0.864			0.658
Median	56 (69.1)	85 (68.0)		50 (70.4)	91 (67.4)	
Low	25 (30.9)	40 (32.0)		21 (29.6)	44 (32.6)	
Surgery			0.468			0.578
Mastectomy	71 (87.7)	105 (84.0)		62 (87.3)	114 (84.4)	
BCS	10 (12.3)	20 (16.0)		9 (12.7)	21 (15.6)	

### Survival prognosis analysis of obesity on TNBC

During the median follow-up of 59 months (range 6–106 months) after diagnosis, 38 deaths and 52 recurrences were documented among 206 TNBC patients included in this analysis. Three cases (1.5 %) were lost to follow-up in the third year after diagnosis, who were neither with obesity nor central obesity.

In univariate analysis of prognostic factors by log-rank test, LVI (DFS: log-rank χ^2^ = 16.864, *P* < 0.001; OS: log-rank χ^2^ = 10.896, *P* = 0.001), pT stage (DFS: log-rank χ^2^ = 15.201, *P* = 0.001; OS: log-rank χ^2^ = 17.619, *P* < 0.001), pN stage (DFS: log-rank χ^2^ = 32.551, *P* < 0.001; OS: log-rank χ^2^ = 33.476, *P* < 0.001), obesity (DFS: log-rank χ^2^ = 3.945, *P* = 0.047; OS: log-rank χ^2^ = 4.113, *P* = 0.043) and central obesity (DFS: log-rank χ^2^ = 3.931, *P* = 0.047; OS: log-rank χ^2^ = 4.987, *P* = 0.026) were significantly predictive of recurrence and survival outcome.

**Fig. 1:**
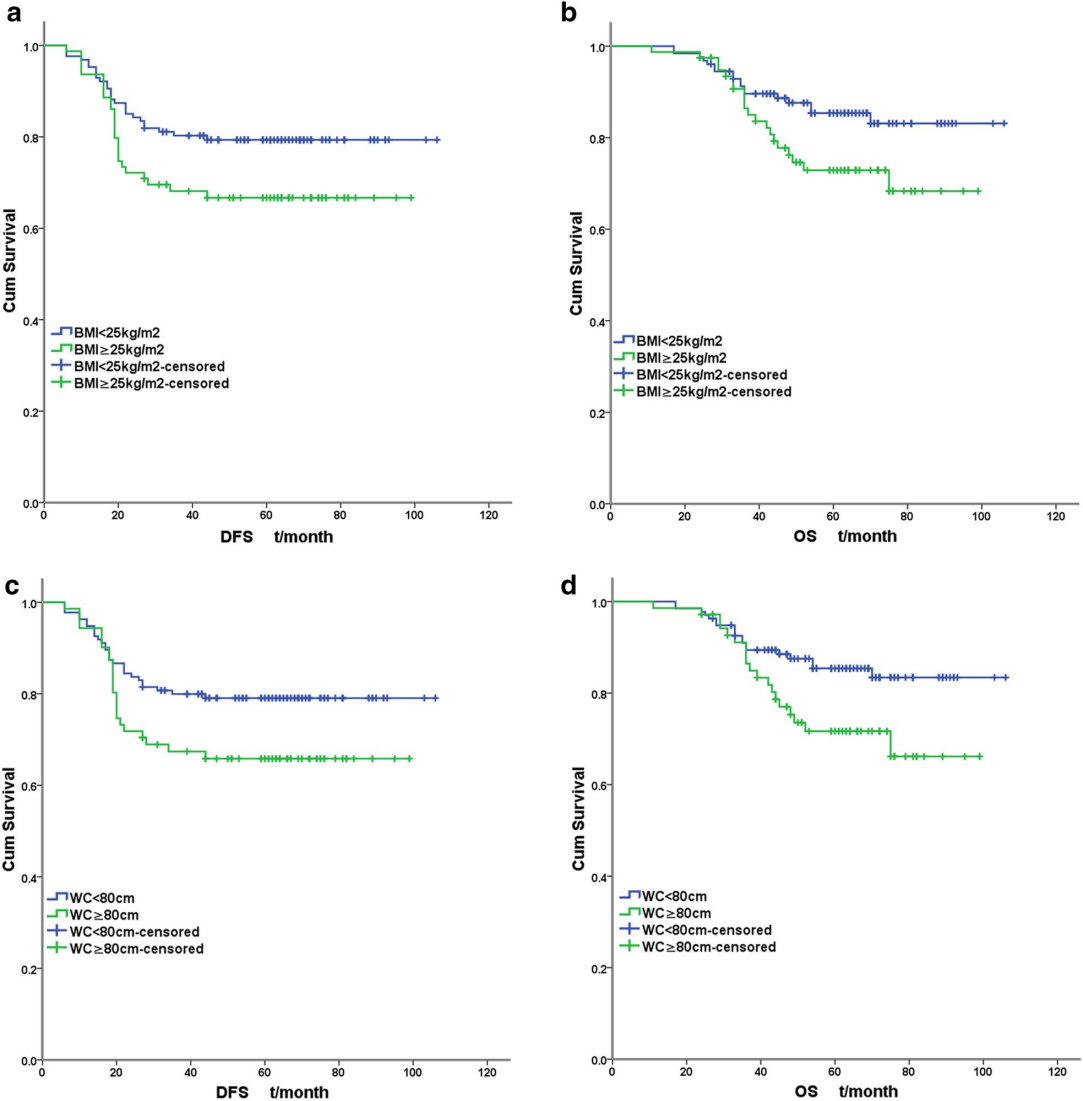
Survival curves of TNBC according to obesity categories. **a** DFS curves according to BMI. **b** OS curves according to BMI. **c** DFS curves according to WC. **d** OS curves according to WC

**Fig. 2:**
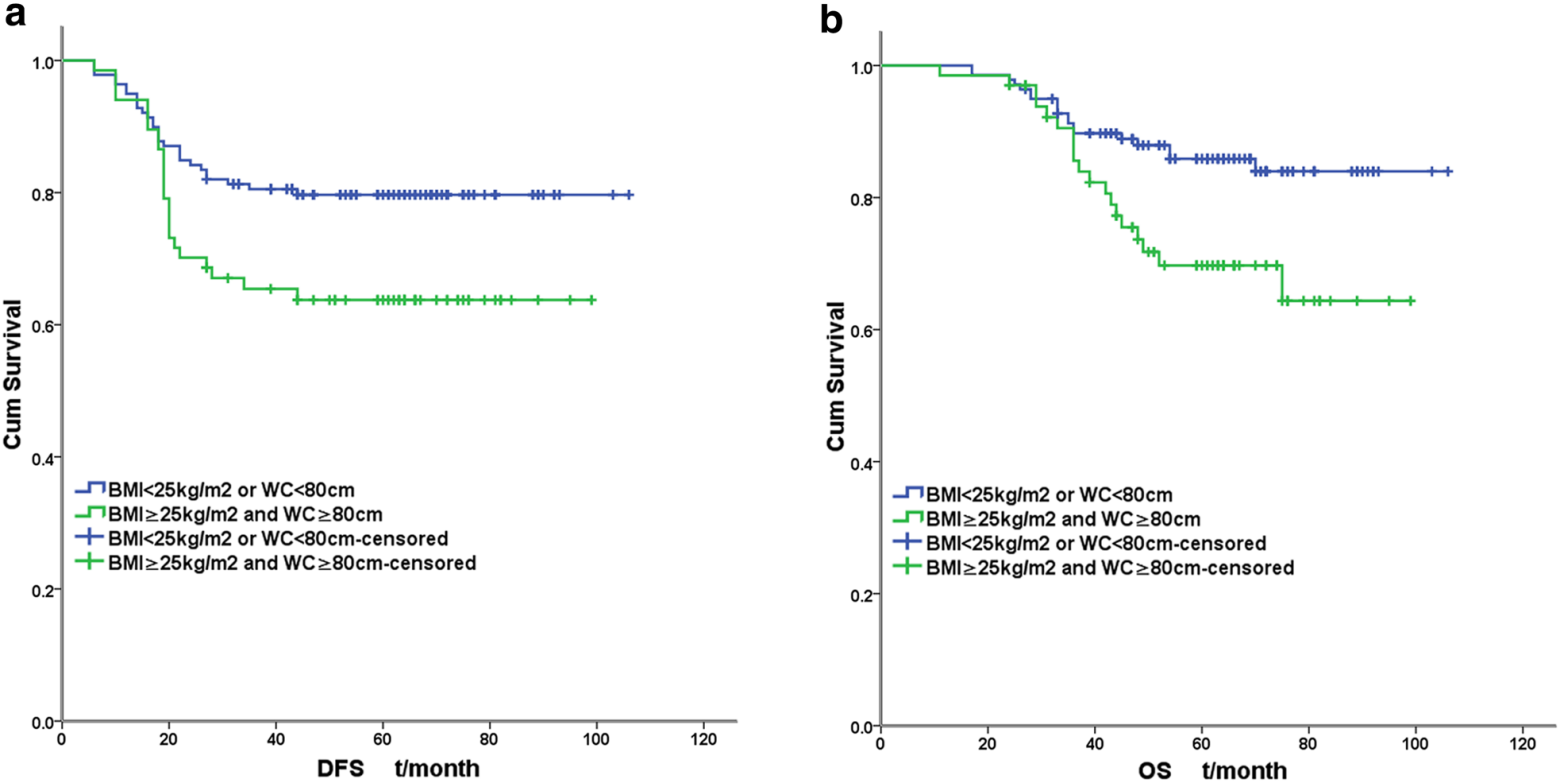
Survival curves of TNBC according to BMI + WC. **a** DFS curves according to BMI + WC. **b** OS curves according to BMI + WC

Figures 1, 2 illustrated the Kaplan–Meier survival curves of obesity categories (obesity, central obesity and central obesity with BMI ≥ 25 kg/m^2^). The separation of survival curves were most apparent among central obesity with BMI ≥ 25 kg/m^2^ (DFS: log-rank χ^2^ = 5.627, *P* = 0.018; OS: log-rank χ^2^ = 6.710, *P* = 0.010).

A Cox regression analysis including the possible prognostic factors above identified pT stage, pN stage, LVI and central obesity (DFS: HR 1.759; 95 % CI 1.009–3.065; *P* = 0.046. OS: HR 2.297; 95 % CI 1.184–4.456; *P* = 0.014.) rather than general obesity (DFS: *P* = 0.125; OS: *P* = 0.059) as independent prognostic factors for DFS and OS of TNBC. For central obesity with BMI ≥ 25 kg/m^2^, the prognostic effect was more apparent (DFS: HR 1.845; 95 % CI 1.059–3.212; *P* = 0.031. OS: HR 2.377; 95 % CI 1.230–4.593; *P* = 0.010.) (Table 2).

**Table 2:** multivariate analysis of TNBC prognostic factors by Cox regression model

	DFS			OS		
	*P*	HR	95.0 % CI	*P*	HR	95.0 % CI
Model 1: BMI ≥ 25 kg/m^2^ as a covariate into Cox regression model						
LVI: positive versus negative	0.012	2.165	1.182–3.965	0.045	2.075	1.017–4.237
pT stage	0.045			0.033		
pT_2_ versus pT_1_	0.037	2.228	1.049–4.736	0.055	2.476	0.981–6.247
pT_3_ versus pT_1_	0.020	3.417	1.217–9.593	0.010	4.672	1.453–15.021
pN stage	0.018			0.048		
pN_1_ versus pN_0_	0.004	2.862	1.410–5.809	0.050	2.304	0.999–5.318
pN_2_ versus pN_0_	0.008	3.016	1.331–6.831	0.009	3.460	1.370–8.736
pN_3_ versus pN_0_	0.108	2.642	0.808–8.636	0.051	3.797	0.993–14.525
BMI: BMI ≥ 25 kg/m^2^ versus BMI < 25 kg/m^2^	0.125	1.554	0.885–2.728	0.059	1.904	0.976–3.713
Model 2: WC ≥ 80 cm as a covariate into Cox regression model						
LVI: positive versus negative	0.011	2.202	1.199–4.043	0.029	2.226	1.083–4.574
pT stage	0.041			0.031		
pT_2_ versus pT_1_	0.030	2.284	1.081–4.826	0.045	2.552	1.020–6.386
pT_3_ versus pT_1_	0.019	3.395	1.218–9.466	0.009	4.635	1.457–14.745
pN stage	0.013			0.033		
pN_1_ versus pN_0_	0.003	2.887	1.419–5.873	0.052	2.303	0.992–5.343
pN_2_ versus pN_0_	0.005	3.231	1.433–7.289	0.005	3.770	1.499–9.477
pN_3_ versus pN_0_	0.107	2.640	0.811–8.589	0.051	3.759	0.994–14.221
WC: WC ≥ 80 cm versus WC < 80 cm	0.046	1.759	1.009–3.065	0.014	2.297	1.184–4.456
Model 3: WC ≥ 80 cm + BMI ≥ 25 kg/m^2^ as a covariate into Cox regression model						
LVI: positive versus negative	0.011	2.207	1.203–4.051	0.037	2.119	1.045–4.294
pT stage	0.041			0.033		
pT_2_:pT_1_	0.031	2.280	1.080–4.814	0.050	2.496	1.001–6.225
pT_3_:pT_1_	0.019	3.394	1.221–9.439	0.010	4.565	1.439–14.488
pN stage	0.017			0.027		
pN_1_:pN_0_	0.004	2.816	1.384–5.730	0.049	2.306	1.002–5.307
pN_2_:pN_0_	0.005	3.159	1.403–7.115	0.004	3.810	1.532–9.476
pN_3_:pN_0_	0.115	2.576	0.793–8.366	0.045	3.850	1.029–14.413
WC + BMI: WC ≥ 80 cm and BMI ≥ 25 kg/m^2^ versus WC < 80 cm or BMI < 25 kg/m^2^	0.031	1.845	1.059–3.212	0.010	2.377	1.230–4.593

## Discussion

Obesity is becoming more and more popular all over the world. According to a recent report, the world has transitioned from one in which underweight prevalence was more than double that of obesity, to one in which more people are obese than underweight. In 2014 there were triple obese men and twice obese women than 40 years ago (NCD Risk Factor Collaboration (NCD-RisC) 2016). The current status of obesity prevalence among Chinese women is also concerning. In 2014, China had the most obese women in the world (46.4 million), accounting for 12.4 % of global obesity (NCD-RisC 2016). A survey in 2015 showed 45.3 % Chinese women were with obesity or overweight (BMI ≥ 24 kg/m^2^), among whom 44.6 % were with central obesity (Wang et al. 2015). In our study, 39.3 % cases were with obesity and 34.5 % cases were with central obesity, which closely mirrored recent trends in the Chinese population.

At the same time, the incidence of breast cancer is rising rapidly in China in recent years. Breast cancer was the most commonly diagnosed cancer among Chinese women in 2015, accounting for 15 % of all new cancers in women (Chen et al. 2016). And breast cancer cases in China account for 12.2 % of all newly diagnosed breast cancers and 9.6 % of all deaths from breast cancer worldwide (Fan et al. 2014). Through a combination of multidisciplinary management, the incorporation of newer and more efficacious chemotherapeutic and biological therapies and implementation of aggressive supportive care services, the prognostic outcome of women with breast cancer has improved over the decades. Women with TNBC continue to have worse prognostic outcomes compared with those with non-TNBC (Dent et al. 2007). Researchers have focused on the mechanisms of TNBC progression and potential targets for therapy. Obesity is a modifiable lifestyle risk factor that has been shown to be associated with increased risk of developing breast cancer including TNBC (Phipps et al. 2011), and is known to predict for the development of distant metastases and breast cancer-related deaths (Ewertz et al. 2011). So it is of great importance to evaluate the impact of obesity on the clinical outcome of TNBC.

Our study showed that obese TNBC tended to be older and to have larger tumor mass, which was in accord with most literature (Ewertz et al. 2011; Deglise et al. 2010). Probably patients with obesity might have difficulty in touching a small lesion in relatively large breast, leading to a larger tumor mass when diagnosed. Deglise et al. (2010) pointed out that infiltrating tumor lesion less than 1 cm was more likely to be impalpable in patients with obesity. It was also reported that post-menopausal TNBC were more frequently observed in patients with obesity (Ewertz et al. 2011; Deglise et al. 2010), which was inconsistent with our study, probably due to not enough post-menopausal cases in our study. Meanwhile our study failed to find the association between obesity and histologic grade, pN stage and LVI of TNBC. The association between obesity and breast cancer characteristics above was also inconsistent among the results from various literatures (Loi et al. 2005; Eichholzer et al. 2013).

A substantial body of evidence exists linking obesity to prognostic outcome among women with breast cancer (Protani et al. 2010; Azrad and Demark-Wahnefried 2014; Chan et al. 2014). A systematic review that included 82 follow-up breast cancer studies showed that obesity was associated with poorer overall and breast cancer survival both in pre-menopausal and post-menopausal breast cancer (Chan et al. 2014).

But only limited research has evaluated the associations of obesity on TNBC prognosis, and the findings are mixed (Ademuyiwa et al. 2011; Sparano et al. 2012; Mowad et al. 2013; Tait et al. 2014; Dawood et al. 2012; Turkoz et al. 2013; Hao et al. 2015). Sparano et al. (2012) observed obesity not to be significantly associated with an inferior prognostic outcome among women with TNBC. The largest retrospective study including 2311 women with stage I-III TNBC tumors found no difference in DFS or OS across BMI groups at diagnosis (Dawood et al. 2012). Similar null association between BMI at diagnosis and TNBC survival was also observed in two retrospective studies of TNBC patients (Ademuyiwa et al. 2011; Tait et al. 2014). While Turkoz et al. (2013) confirmed the relationship between obesity and poorer prognostic outcome of TNBC. And a study on Chinese women also drew a conclusion that BMI ≥ 24 kg/m^2^ was a negative prognostic factor of TNBC (Hao et al. 2015).

The difference in the study design, the characteristics of study population, duration of follow-up or cut-off value of BMI assessment may be partly contributed to the discrepancy. Most research defined obesity as BMI ≥ 30 kg/m^2^ according to the World Health Organization guideline, which was based on the body size characteristics of western country population. This standard did not fit for Chinese population. As a growing body of evidence suggested a strong link between metabolic syndrome and breast cancer, BMI ≥ 25 kg/m^2^ as obesity standard by international diabetics federation was adopted in our study. It could be observed that a large proportion of TNBC patients with central obesity in our study had some kinds of cardiovascular or metabolic disease.

Our study was the first to specifically evaluate the prognostic outcome effect of central obesity on TNBC. It was concluded that BMI ≥ 25 kg/m^2^ was not an independent prognostic factor of TNBC although it was of statistical significance in univariate analysis. But central obesity was the independent prognostic factor of TNBC, and the effect was even more apparent in central obesity with BMI ≥ 25 kg/m^2^. Probably WC may mistake some tall and strong women as obesity, although which was rarely observed in Chinese women. The implementation of BMI could distinguish TNBC with poorer prognosis even better.

A recent Spanish study pointed out that almost half of the obese population were metabolically healthy obese phenotype (Goday et al. 2016). But central obesity is a key factor in metabolic syndrome, which is linked to the development of diabetes and cardiovascular disease and is also closely related with the development of breast cancer, especially TNBC (Davis and Kaklamani 2012; Maiti et al. 2010). Systematic reviews indicated that central obesity added 79 % risk of breast cancer among premenopausal women and 50 % among postmenopausal women (Connolly et al. 2002; Harvie et al. 2003). And central obesity was associated with poorer outcome of breast cancer as well (Abrahamson et al. 2006).

Central obesity is characterized by the accumulation of visceral fat and WC is a correlate of the amounts of visceral fat. There were apparent differences between visceral fat and subcutaneous fat with regard to receptor distribution, factors secreted by adipose tissue and enzyme activity in adipose cell. Compared with subcutaneous fat, visceral fat have more blood supply and nerve distribution and are more likely to accumulate and decompose. Visceral fat is more metabolically active.

General obesity is linked to elevated levels of estrogen among post-menopausal women. The correlation between general obesity and poorer prognosis of breast cancer may be mediated by increased circulating estrogen levels from excess adiposity through aromatase activity and reduced levels of sex hormone-binding globulins (Rose and Vona-Davis 2009). Such mechanism is more common in postmenopausal obesity patients with positive hormone receptor. In contrast, the main mechanism of central obesity promoting TNBC progression is the disturbance of the ‘insulin-leptin-adiponectin’ axis. Central obesity is an independent predictor of insulin resistance (Seidell et al. 1990) and higher levels of free insulin-like growth factor-1 (IGF-1) (Lukanova et al. 2001) compared with general obesity. Increased level of insulin and IGF-1 in central obesity were mitogenic agents and promoted breast cancer cell proliferation directly (Azrad and Demark-Wahnefried 2014; Demark-Wahnefried et al. 2012). They could also accelerate tumor cell growth and migration by activating tumor neovascularization (Azrad and Demark-Wahnefried 2014). Leptin and adiponectin are both obesity related regulatory proteins secreted by adipose tissue. Leptin increases in obesity. It could enhance IGF-1 receptor activity and promote TNBC cell proliferation and migration. It could also promote survival of cancer stem cells in vivo, consequently promoting breast cancer (Zheng et al. 2013; Oh et al. 2011). Meanwhile, the level of adiponectin is low in obese patients. Adiponectin is the intrinsic insulin sensitizer and plays an antitumor effect opposite to leptin. Adiponectin could inhibit hormone receptor negative breast cancer cell proliferation and induce apoptosis through various signal transduction pathways (Jardé et al. 2011). Moreover, the abundant of inflammation cytokines secreted by activated macrophages in the adipose tissue, such as TNFα, IL-6, etc., constitutes tumor microenvironment, which promotes tumor cell migration and invasion (Howe et al. 2013).

It should be noted that our study has some limitations, including the retrospective nature of the study design, relatively small sample size, short follow-up duration, and lack of information on longitudinal change in BMI after breast cancer diagnosis which is known to be of prognostic value (Kroenke et al. 2005; Demark-Wahnefried et al. 2001).

## Conclusion

In conclusion, this single-institution study indicated that central obesity, especially with high BMI, was an independent prognostic factor of OS or DFS in patients with TNBC. It was the first study focusing on the association between central obesity and TNBC outcome. While the interest pertaining to the association between TNBC outcomes and obesity should be warranted in larger prospective studies, TNBC patients should be counseled on maintaining a healthy weight as a manner of treatment.
